# Supplementary material for: Post-catalysis structures of mitochondrial complex I with ubiquinol-10 bound in the active site
Source: Nat Commun. 2026 Mar 5;17:3506. doi: 10.1038/s41467-026-70030-0 (PMC13083836; doi:10.1038/s41467-026-70030-0)
Supplement: Supplementary file 1 — Supplementary Information [file 41467_2026_70030_MOESM1_ESM.pdf]

Supplementary Information

Post-catalysis structures of mitochondrial complex I with ubiquinol-10 bound in the active site

Injae Chung, Caroline S. Pereira, John J. Wright, Guilherme M. Arantes*, Judy Hirst*

* Address correspondence to:

Guilherme M. Arantes, Department of Biochemistry, Instituto de Química, Universidade de São Paulo, Av. Prof. Lineu Prestes 748, 05508-900, São Paulo, SP, Brazil

e-mail: garantes@iq.usp.br

Judy Hirst, MRC Mitochondrial Biology Unit, University of Cambridge, The Keith Peters Building, Cambridge Biomedical Campus, Hills Road, Cambridge, CB2 0XY, UK

e-mail: jh480@cam.ac.uk

This file includes:

Supplementary Figures 1-7

Supplementary Tables 1 and 2

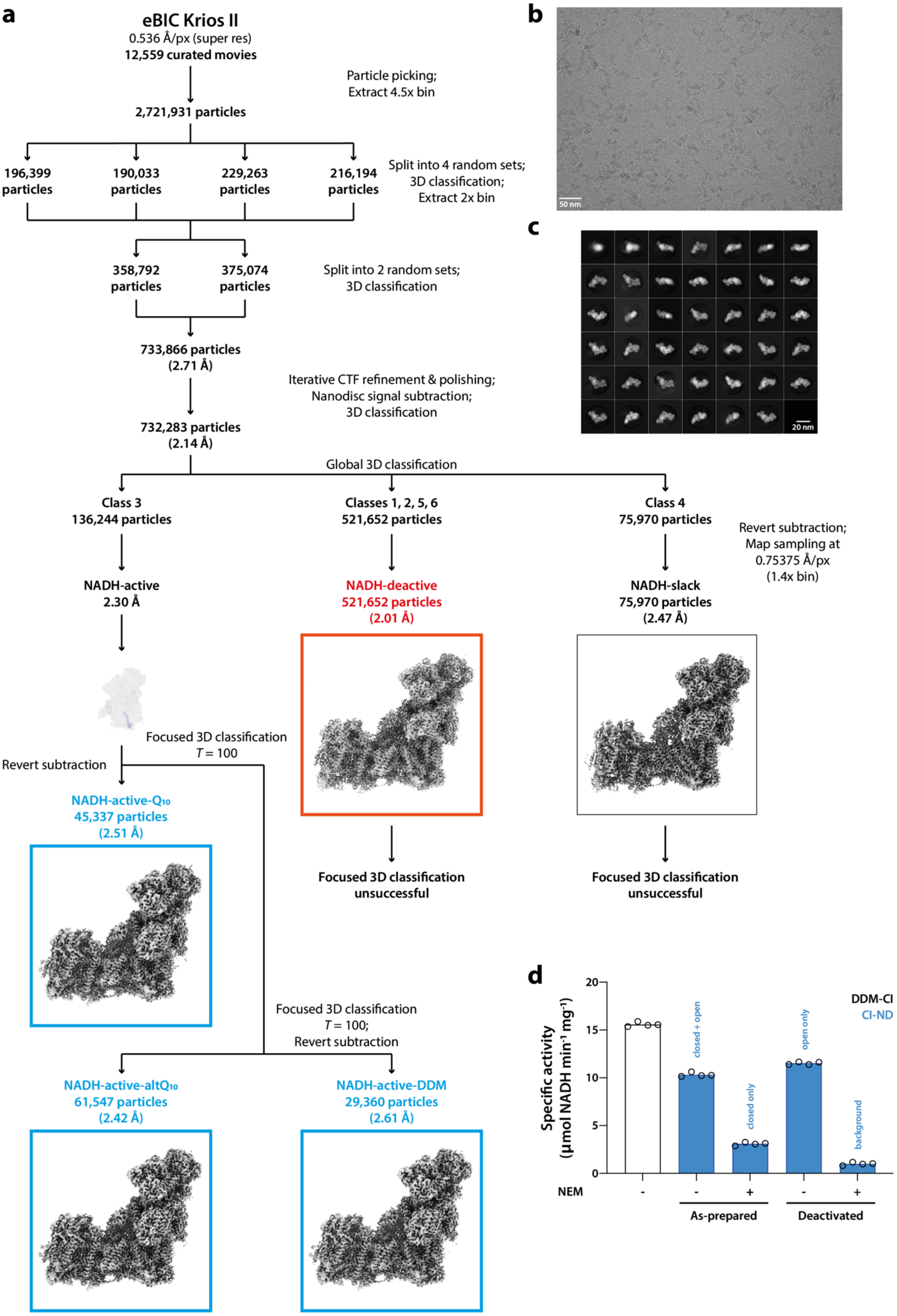
a
eBIC Krios II
0.536 Å/px (super res)
12,559 curated movies
Particle picking;
Extract 4.5x bin
2,721,931 particles
196,399 particles
190,033 particles
229,263 particles
216,194 particles
Split into 4 random sets;
3D classification;
Extract 2x bin
358,792 particles
375,074 particles
Split into 2 random sets;
3D classification
733,866 particles
(2.71 Å)
Iterative CTF refinement & polishing;
Nanodisc signal subtraction;
3D classification
732,283 particles
(2.14 Å)
Global 3D classification
Class 3
136,244 particles
Classes 1, 2, 5, 6
521,652 particles
Class 4
75,970 particles
Revert subtraction;
Map sampling at
0.75375 Å/px
(1.4x bin)
NADH-active
2.30 Å
NADH-deactive
521,652 particles
(2.01 Å)
NADH-slack
75,970 particles
(2.47 Å)
Focused 3D classification
T = 100
Revert subtraction
NADH-active-Q10
45,337 particles
(2.51 Å)
Focused 3D classification
unsuccessful
Focused 3D classification
unsuccessful
Focused 3D classification
T = 100;
Revert subtraction
NADH-active-altQ10
61,547 particles
(2.42 Å)
NADH-active-DDM
29,360 particles
(2.61 Å)
b
50 nm
c
20 nm
d
Specific activity
(µmol NADH min⁻¹ mg⁻¹)
20
15
10
5
0
DDM-CI
CI-ND
closed + open
closed only
open only
background
NEM
-
-
+
-
+
As-prepared
Deactivated

Supplementary Figure 1: Cryo-EM data processing. (**a**) A flow chart of cryo-EM data processing leading to five distinct classes. Blue, red, and black boxes denote the final maps for active, deactive, and slack classes, respectively. (**b**) A representative micrograph. (**c**) Representative 2D averages. The example view was selected following 2D classification of the final 3D refined particles to show classes of particles in different orientations. (**d**) NADH:dQ oxidoreductase activities of DDM-solubilised isolated complex I (DDM-CI; white) and CI-ND (blue) in the presence of CHAPS/asolectin (to dissociate the MSP2N2 and provide a larger hydrophobic phase). The proportion of the active/closed and deactive/open states in as-prepared and deactivated CI-ND samples was checked by the sensitivity of catalysis to derivatisation with NEM, relative to the background rate. 22.4% of the as-prepared enzyme was found to be in the active/closed state, using the specific activity of NEM-treated, deactivated CI-ND (1.0 μmol min^{-1} mg^{-1}) as the background rate. The equivalent value calculated from the particle numbers is 20.7%. All data are mean averages from four technical replicates.

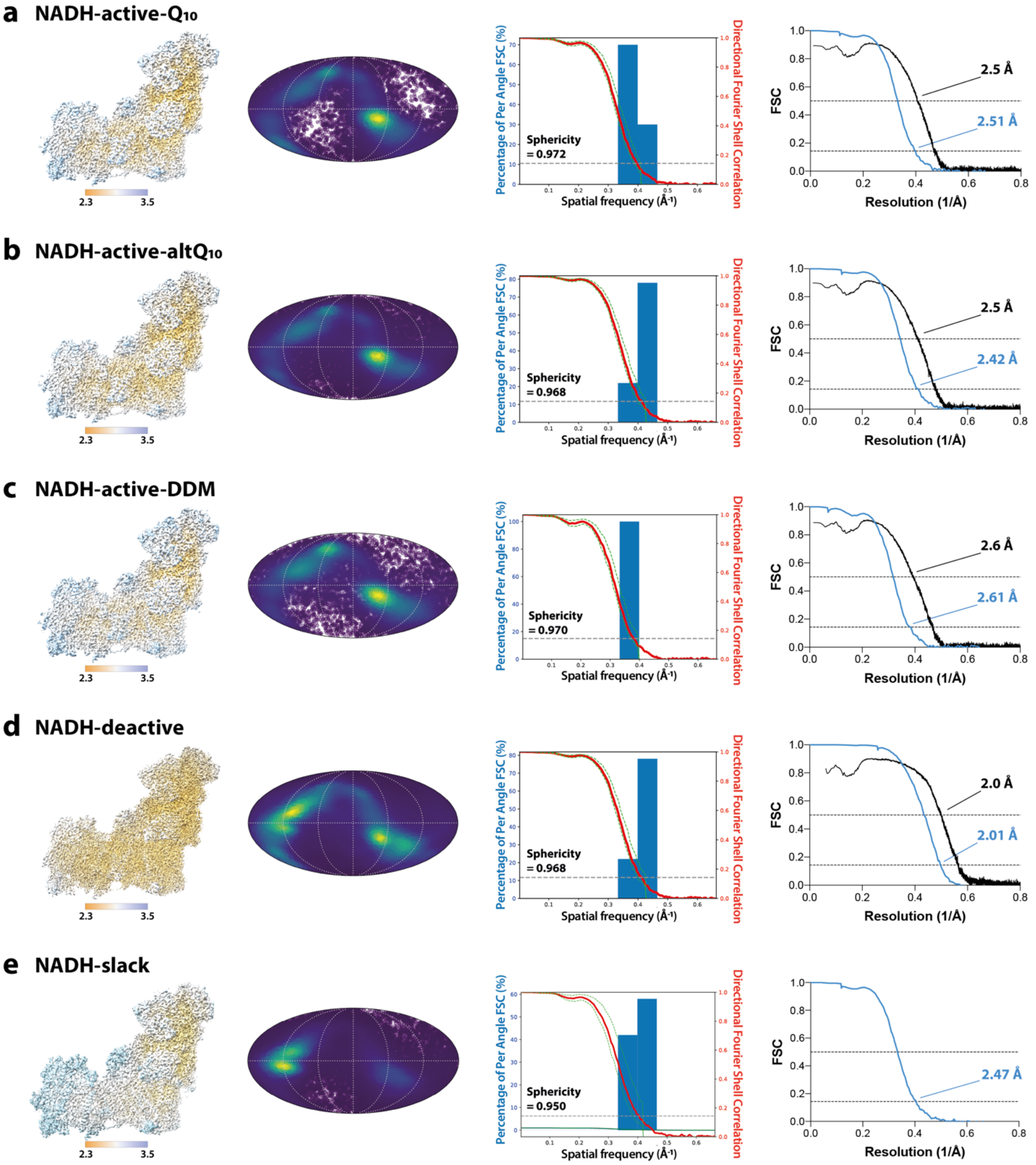

Supplementary Figure 2: Local resolution maps, Mollweide projections, *3DFSC* plots, and Fourier shell correlation curves for the five NADH-CI-ND states. Local resolution consensus maps (left), Mollweide projections (middle left), histogram and directional FSC (*3DFSC*[62]) plots (middle right), and Fourier shell correlation (FSC) curves (right) are shown for the (**a**) NADH-active-Q_{10}, (**b**) NADH-active-altQ_{10}, (**c**) NADH-active-DDM, (**d**) NADH-deactive, and (**e**) NADH-slack structures. Local resolutions were estimated using the *Local Resolution* function in RELION[59] and plotted using UCSF ChimeraX[55] with map thresholds (σ) of 6.5, 6.5, 6.5, 4.5, and 5.0, respectively. Coloured keys indicate resolution in Å. Mollweide projections were plotted using Python and *Matplotlib*, and the degree of directional resolution anisotropy

(termed *Sphericity*) calculated using the 3DFSC program suite[62]. Blue bars represent histogram of directional FSCs, red line indicates global FSC, and green dashed lines indicate ± 1 standard deviation from mean of directional FSC. RELION half-map (sky blue) and model-map (black, where relevant) FSC curves are shown.

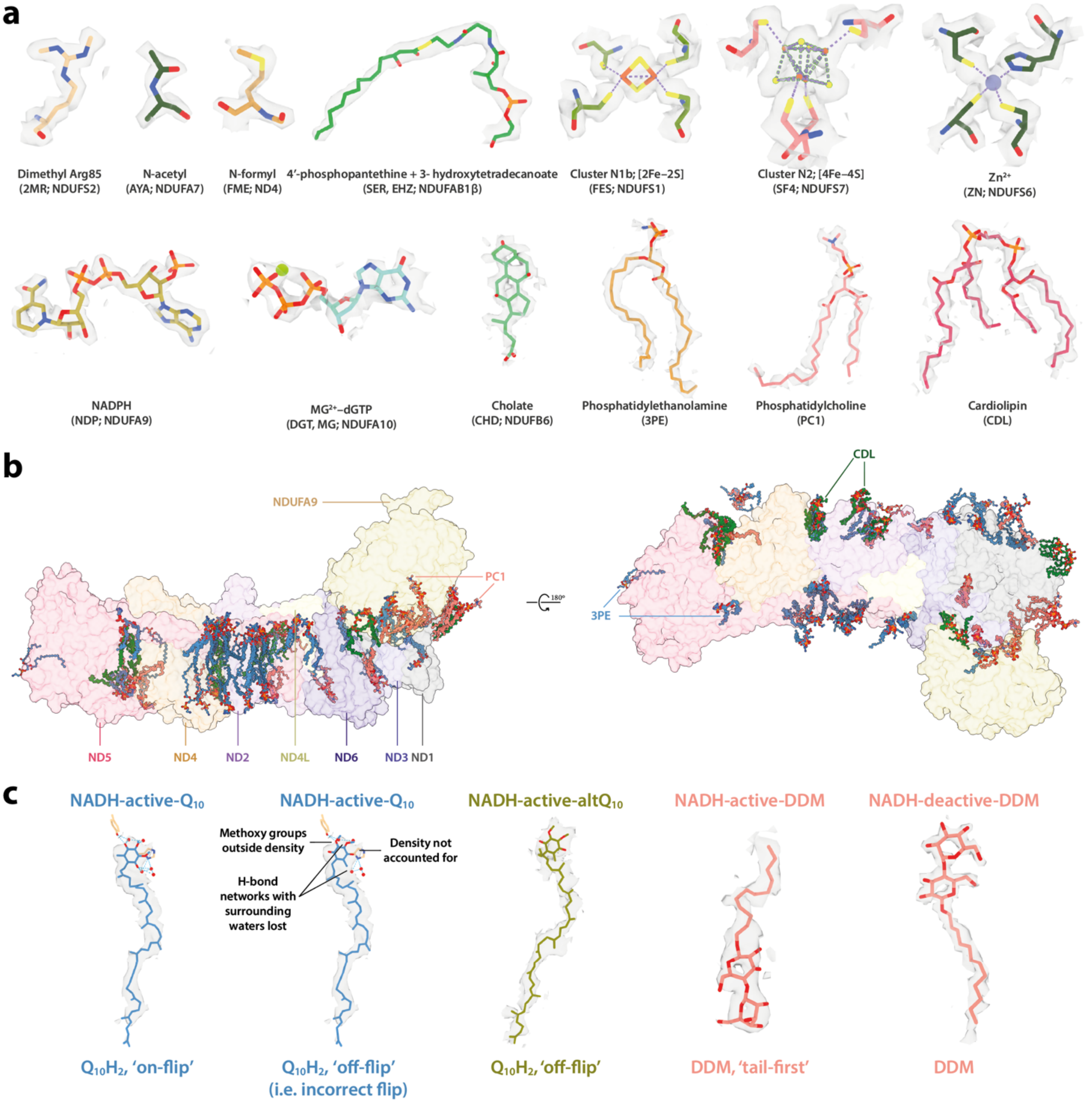

Supplementary Figure 3: Modelled ligands, ions, lipids, and modified residues. (**a**) A non-exhaustive array of ligands, lipid, and modified residues modelled in the NADH-deactive structure, the highest resolution cryo-EM map in this study (global resolution of 2.0 Å). Densities are shown at map thresholds between 1.5 to 4.0 in UCSF ChimeraX[55]. (**b**) Side (left) and top (right) views of all NADH-active and deactive models superimposed on subunit ND5, showing modelled phosphatidylethanolamine (3PE, blue), phosphatidylcholine (PC1, salmon), and cardiolipin (CDL, green) molecules. The seven conserved core membrane subunits and NDUFA9 are labelled in transparent colour. (**c**) Densities for $Q_{10}H_2$ and DDM in the Q-binding sites of NADH-active and -deactive maps are shown at the following map thresholds (σ) in UCSF ChimeraX: NADH-active-Q_{10}, 5.0; NADH-active-altQ_{10}, 3.0; NADH-active-DDM, 5.9; NADH-deactive-DDM, 2.5. For NADH-active-Q_{10}, on- and off-flip poses are shown alongside Tyr108^{NDUFS2}, His59^{NDUFS2}, and surrounding water molecules. Hydrogen bonds are shown as blue dashed lines.

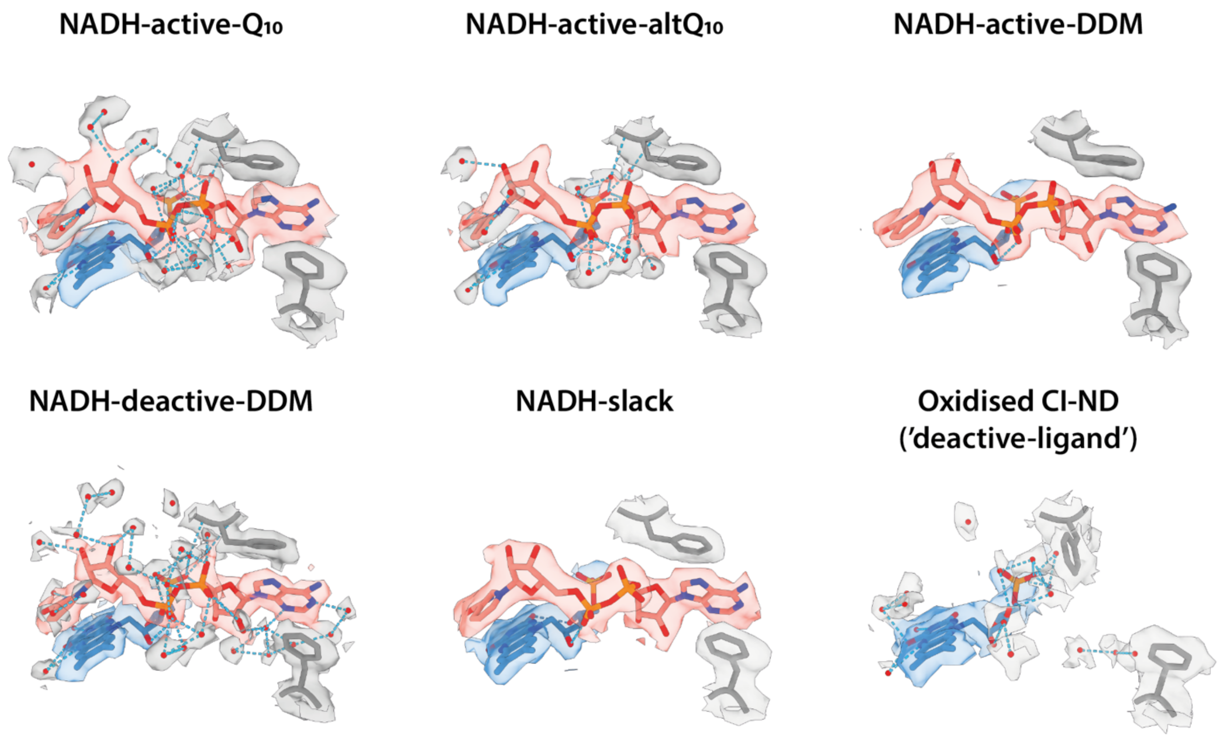

Supplementary Figure 4: NADH binding site in active, deactive, and slack CI-ND maps. (**a**) Densities for NADH, FMN, Phe73^{NDUFV1}, Phe209^{NDUFV1}, and surrounding waters (<5 Å; red spheres) in the flavin site of all five NADH-bound maps resolved in this study and a representative, highest resolution oxidised CI-ND map determined in our previous study[8], displayed in matching views. Water molecules were not modelled in NADH-active-DDM and NADH-slack. PDB-7QSM and EMD-14134 (ref. [8]) were used to make the 'Oxidised CI-ND' panel. Map densities are shown in blue (FMN), salmon (NADH), or grey (protein and waters), at the following map thresholds (σ) in UCSF ChimeraX[55]: NADH-active-Q_{10}, 4; NADH-active-altQ_{10}, 5; NADH-active-DDM, 6.5; NADH-deactive-DDM, 2; NADH-slack, 5; Oxidised CI-ND, 1.5. Hydrogen bonds are shown as blue dashed lines.

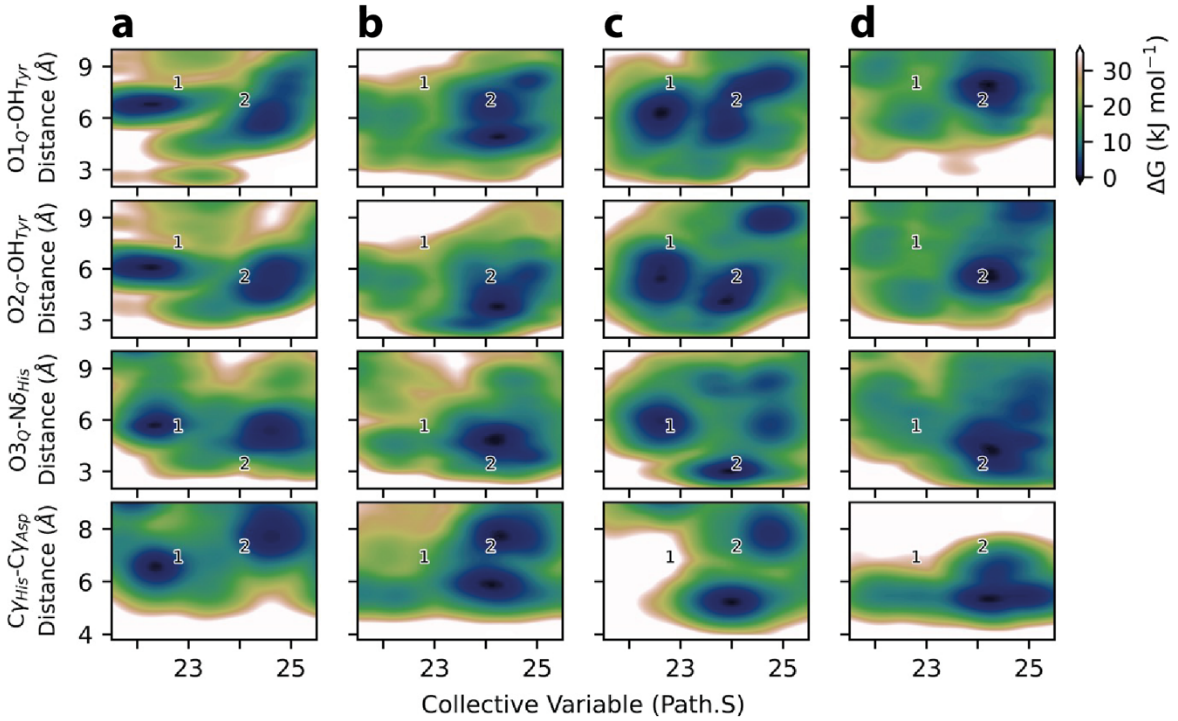

Supplementary Figure 5: Free energy profiles for additional structural properties obtained from molecular simulations. Four combinations of sidechain protonation were simulated (Figure 3 in main text): **a**, [Tyr-, His, Asp-] (total charge -2); **b**, [Tyr, His, Asp-] (total charge -1); **c**, [Tyr, His, AspH] (total charge 0); and **d**, [Tyr, HisH+, Asp-] (total charge 0). Properties correspond to atom-pair distances indicated on the left. Numbers correspond to pairs of Path.S and distances observed in the NADH-active-Q_{10} (1) and NADH-active-altQ_{10} (2) cryo-EM models. The [Tyr-, His, Asp] charge-state displays free energy minima close (within 5 kJ mol^{-1}) to distances observed in the NADH-active-Q_{10} cryo-EM model (1) for all structural properties.

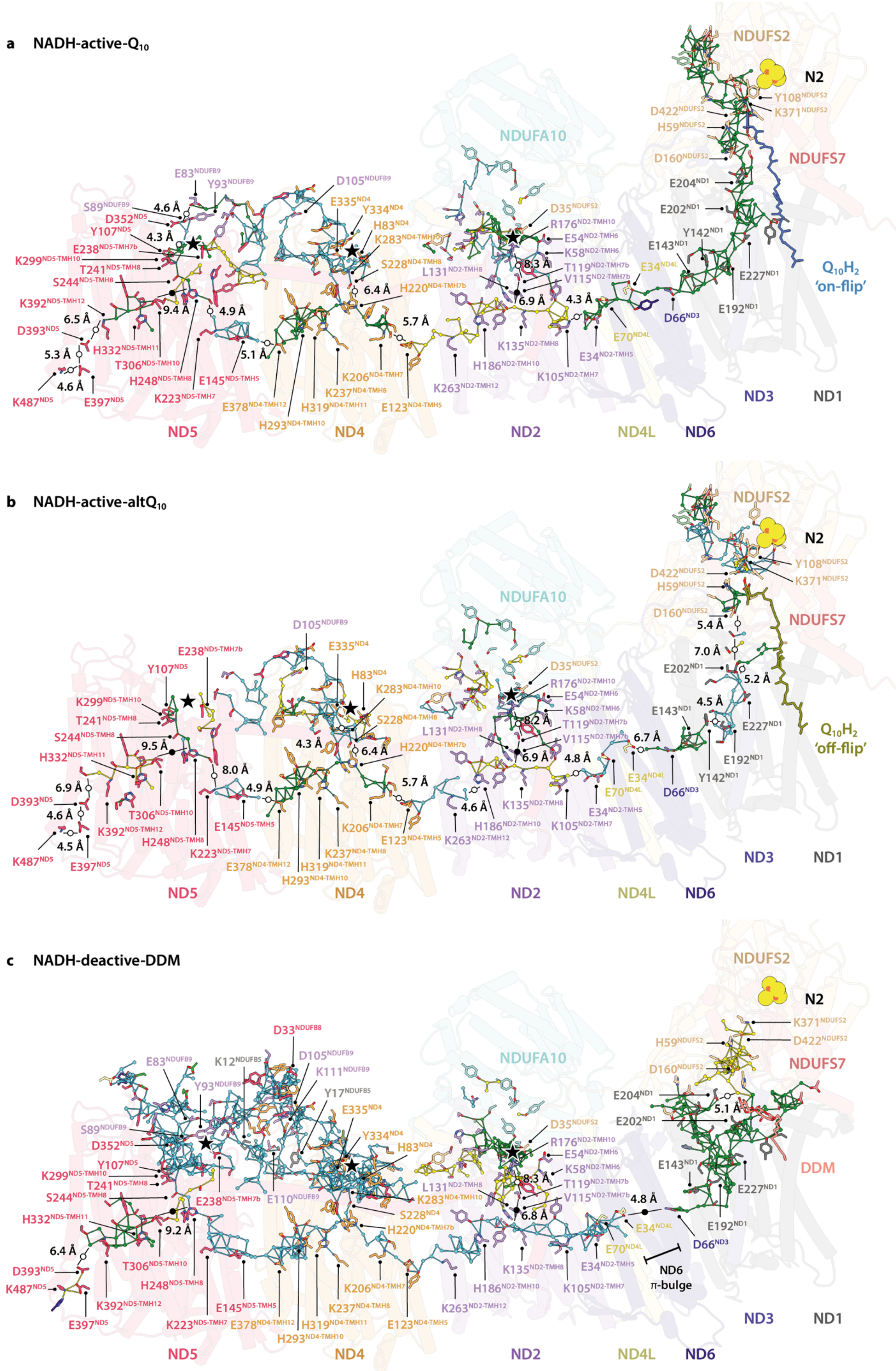

a NADH-active-Q_{10}
NDUFS2
N2
Y108 NDUFS2
K371 NDUFS2
D422 NDUFS2
H59 NDUFS2
D160 NDUFS2
NDUFS7
NDUFA10
E204 ND1
E202 ND1
Y142 ND1
E143 ND1
E227 ND1
E192 ND1
$Q_{10}H_2$ 'on-flip'
E83 NDUFB9
Y93 NDUFB9
S89 NDUFB9
D352 ND5
Y107 ND5
E238 ND5-TMH7b
K299 ND5-TMH10
T241 ND5-TMH8
S244 ND5-TMH8
K392 ND5-TMH12
D393 ND5
H332 ND5-TMH11
T306 ND5-TMH10
H248 ND5-TMH8
E145 ND5-TMH5
K223 ND5-TMH7
K487 ND5
E397 ND5
D105 NDUFB9
E335 ND4
Y334 ND4
H83 ND4
K283 ND4-TMH10
S228 ND4-TMH8
H220 ND4-TMH7b
K206 ND4-TMH7
K237 ND4-TMH8
E378 ND4-TMH12
H319 ND4-TMH11
H293 ND4-TMH10
E123 ND4-TMH5
D35 NDUFS2
R176 ND2-TMH10
E54 ND2-TMH6
K58 ND2-TMH6
L131 ND2-TMH8
T119 ND2-TMH7b
V115 ND2-TMH7b
K135 ND2-TMH8
H186 ND2-TMH10
K263 ND2-TMH12
K105 ND2-TMH7
E34 ND2-TMH5
E34 ND4L
E70 ND4L
D66 ND3
4.6 Å
4.3 Å
9.4 Å
4.9 Å
6.5 Å
5.3 Å
4.6 Å
5.1 Å
6.4 Å
5.7 Å
8.3 Å
6.9 Å
4.3 Å
ND5
ND4
ND2
ND4L
ND6
ND3
ND1
b NADH-active-altQ_{10}
NDUFS2
N2
Y108 NDUFS2
K371 NDUFS2
D422 NDUFS2
H59 NDUFS2
D160 NDUFS2
NDUFS7
NDUFA10
5.4 Å
7.0 Å
E202 ND1
5.2 Å
4.5 Å
E143 ND1
E227 ND1
E192 ND1
Y142 ND1
$Q_{10}H_2$ 'off-flip'
D105 NDUFB9
E238 ND5-TMH7b
Y107 ND5
K299 ND5-TMH10
T241 ND5-TMH8
S244 ND5-TMH8
H332 ND5-TMH11
9.5 Å
8.0 Å
6.9 Å
D393 ND5
4.6 Å
T306 ND5-TMH10
K392 ND5-TMH12
H248 ND5-TMH8
4.5 Å
K487 ND5
E397 ND5
K223 ND5-TMH7
E145 ND5-TMH5
4.9 Å
E335 ND4
H83 ND4
K283 ND4-TMH10
S228 ND4-TMH8
4.3 Å
6.4 Å
H220 ND4-TMH7b
5.7 Å
K206 ND4-TMH7
E123 ND4-TMH5
K237 ND4-TMH8
E378 ND4-TMH12
H319 ND4-TMH11
H293 ND4-TMH10
4.6 Å
H186 ND2-TMH10
K263 ND2-TMH12
D35 NDUFS2
R176 ND2-TMH10
E54 ND2-TMH6
K58 ND2-TMH6
L131 ND2-TMH8
8.2 Å
T119 ND2-TMH7b
V115 ND2-TMH7b
6.9 Å
4.8 Å
6.7 Å
K135 ND2-TMH8
K105 ND2-TMH7
E34 ND2-TMH5
E34 ND4L
E70 ND4L
D66 ND3
ND5
ND4
ND2
ND4L
ND6
ND3
ND1
c NADH-deactive-DDM
NDUFS2
N2
K371 NDUFS2
D422 NDUFS2
H59 NDUFS2
D160 NDUFS2
NDUFS7
NDUFA10
E204 ND1
5.1 Å
E202 ND1
E143 ND1
E227 ND1
E192 ND1
DDM
D33 NDUFB8
E83 NDUFB9
K12 NDUFB5
D105 NDUFB9
K111 NDUFB9
Y93 NDUFB9
Y17 NDUFB5
E335 ND4
S89 NDUFB9
D352 ND5
Y334 ND4
H83 ND4
Y107 ND5
K299 ND5-TMH10
T241 ND5-TMH8
S244 ND5-TMH8
E238 ND5-TMH7b
E110 NDUFB9
L131 ND2-TMH8
K283 ND4-TMH10
S228 ND4
H220 ND4-TMH7b
H332 ND5-TMH11
9.2 Å
6.4 Å
T306 ND5-TMH10
H248 ND5-TMH8
D393 ND5
K487 ND5
K392 ND5-TMH12
E397 ND5
E145 ND5-TMH5
K223 ND5-TMH7
E378 ND4-TMH12
H319 ND4-TMH11
H293 ND4-TMH10
K206 ND4-TMH7
K237 ND4-TMH8
E123 ND4-TMH5
K263 ND2-TMH12
D35 NDUFS2
R176 ND2-TMH10
E54 ND2-TMH6
K58 ND2-TMH6
8.3 Å
T119 ND2-TMH7b
V115 ND2-TMH7b
6.8 Å
4.8 Å
E34 ND4L
D66 ND3
E70 ND4L
ND6 π-bulge
K135 ND2-TMH8
H186 ND2-TMH10
E34 ND2-TMH5
K105 ND2-TMH7
ND5
ND4
ND2
ND4L
ND6
ND3
ND1

Supplementary Figure 6: Candidate proton-transfer networks in the ubiquinone-binding site and membrane domain of three states of complex I in NADH-CI-NDs. (**a**-**c**) Side views of the networks in the ubiquinone-binding site, the E-channel, and the central axis of (**a**) NADH-active-Q_{10}, (**b**) NADH-active-altQ_{10}, and (**c**) NADH-deactive-DDM. Relevant Grotthuss-competent residues (Asp, Glu, His, Lys, Ser, Thr, and Tyr) and water molecules are shown in stick and sphere representations; for each proposed Grotthuss network the links are coloured differently (green, yellow, and blue). Only key residues are labelled. Gaps in the networks are denoted by black lines, with distances displayed and solid and open circles highlighting protein-obstructed and unobstructed gaps, respectively. Matrix-side entry points (in the half-channels of the antiporter-like subunits) are indicated (★). Networks were detected using a cut-off distance of 4 Å between the protonatable N and O centres[10,11,21,43].

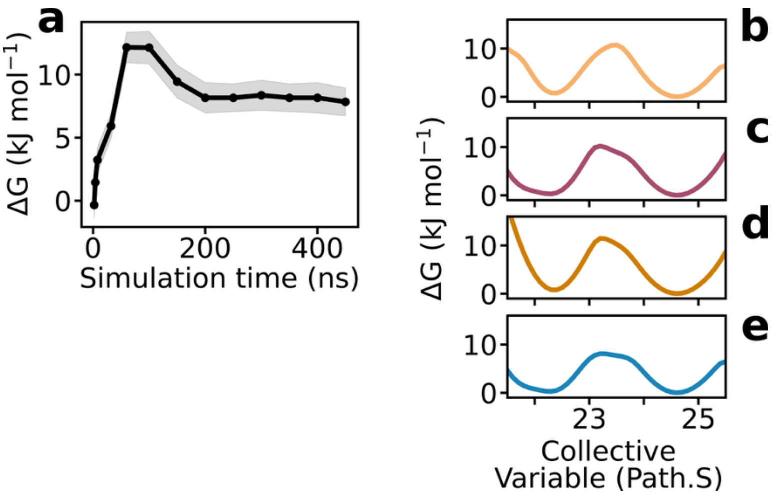

Supplementary Figure 7: Convergence of the computed free energy profiles. The analysis is for simulations for the [Tyr-, His, Asp-] protonation state (Figure 3**a**). (**a**) Convergence of the free energy (ΔG) barrier with simulation time, computed as the difference between Path.S = 22.3 and Path.S = 23.2. The statistical uncertainty from a bootstrap analysis is shown as the shaded region. The change in the free energy difference becomes smaller than the statistical uncertainty after ~150 ns, indicating convergence. (**b–d)** Free energy profiles obtained by splitting the full trajectory into three consecutive segments of 150 ns each. (**e**) Profile obtained from the complete 450 ns trajectory (the same as shown in Figure 3**a**, reproduced for comparison). The similar overall shapes and relative free energies across the profiles (with standard deviations <1.3 kJ mol^{-1}, except in the less sampled region with Path.S < 22.0 that are not visited experimentally) support the convergence of the simulation and that the results are not sensitive to the initial configuration.

Supplementary Table 1: Cryo-EM data collection, refinement, and validation statistics

			NADH-CI-ND<br>EMPIAR-13115		
**Data Collection and Processing**					
Magnification			81,000		
Voltage (kV)			300		
Electron exposure (e^{-}/Å^{2})			40.29		
Defocus range (µm)			-0.9 to -2.3		
Super-resolution pixel size (Å)			0.536		
Final pixel size (Å)			0.75375		
Symmetry imposed			C1		
Initial particle images (no.)			2,721,931		
Final particle images (no.)			733,866		
**Classes**	**NADH Active Q_{10}**	**NADH Active AltQ_{10}**	**NADH Active DDM**	**NADH Deactive (DDM)**	**NADH Slack (Cholate)**
**EMDB ID**	**EMD-55030**	**EMD-55031**	**EMD-55032**	**EMD-55033**	**EMD-55034**
**PDB ID**	**9SMF**	**9SMG**	**9SMH**	**9SMI**	**-**
Final particle images (no.)	45,337	61,547	29,360	521,652	75,970
Map resolution (Å)<br>FSC threshold: 0.143	2.51	2.42	2.61	2.01	2.47
Map resolution range (Å)	2.28–7.02	2.21–5.93	2.33–7.27	1.84–4.73	2.16–6.28
Map sharpening *B*-factor (Å^{2})	-11	-12	-12	-44	-28
**Model Statistics**					
Initial model (PDB ID)	7QSK	7QSK	7QSK	7QSM	7QSO
Model resolution (Å)<br>FSC threshold: 0.5	2.85	2.73	2.23	2.23	
Model composition					
Non-hydrogen atoms	70,931	70,726	68,600	72,104	
Protein residues	8,299	8,299	8,285	8,219	
Ligands	48	45	44	48	
Waters	1,997	1,971	–	3,797	
Average *B* factors (Å^{2})					
Protein	40.44	41.79	34.39	24.12	
Ligand	51.47	52.37	42.03	32.65	
Water	33.00	35.09	–	26.81	
Root Mean Square deviations					
Bond lengths (Å)	0.003	0.004	0.002	0.002	
Bond angles (°)	0.944	0.980	0.533	0.525	
MolProbity score	1.10	1.01	1.00	1.06	
All-atom clash score	3.11	2.29	2.26	2.75	
EMRinger score	4.92	4.86	5.05	6.12	
Rotamer outliers (%)	0.51	0.19	0.87	0.81	
Ramachandran plot					
Favoured (%)	98.05	98.14	98.14	98.15	
Allowed (%)	1.93	1.78	1.81	1.79	
Outliers (%)	0.02	0.07	0.05	0.06	
Rama-Z score, RMSD					
whole	1.04	0.85	0.99	1.16	
helix	1.25	1.05	1.19	1.30	
sheet	0.00	0.11	0.00	0.18	
loop	0.51	0.17	0.20	0.30	

Supplementary Table 2: Additional information on experimental structures of closed CI with bound Q species. The models are the same as in Table 2. Atom-pair distances are in Å. Hydrogen bond donor-acceptor distances are shown only when the bonds fulfil the expected geometric criteria.

Model	**1**	**2**	**3**	**4**	**5**	**6**	**7**	**8**	**9**	**10**	**11**	**12**	**13**
PDB	Fig. 2c (left, right)		7QSK	8Q48	8Q45	7V2C	7V2R	7V2H	8UEP	6ZKC	8ESZ	7Z7S	7Z80
$C6_Q$-$C\beta_{Tyr}$	10.3	13.1	12.0	10.1	10.1	10.3	9.9	10.9	10.6	10.9	10.1	11.4	11.7
$C\gamma_{His}$-$C\gamma_{Asp}$	6.9	7.3	7.2	6.6	6.7	5.5	5.5	7.0	7.1	6.1	6.4	6.8	7,0
H-bonds:													
$O4_Q$-Tyr	2.8			2.5	2.7	3.2	3.0				-		
$O4_Q$-His										2.8			
$O2/3_Q$-Tyr								3.3					
$O2/3_Q$-His			3.2			2.9				2.6	3,0		
His-Asp						2.6	2.6						
